# Effects of Inclined Interface Angle on Compressible Rayleigh–Taylor Instability: A Numerical Study Based on the Discrete Boltzmann Method

**DOI:** 10.3390/e25121623

**Published:** 2023-12-05

**Authors:** Bailing Chen, Huilin Lai, Chuandong Lin, Demei Li

**Affiliations:** 1 School of Mathematics and Statistics, Key Laboratory of Analytical Mathematics and Applications (Ministry of Education), Fujian Key Laboratory of Analytical Mathematics and Applications (FJKLAMA), Center for Applied Mathematics of Fujian Province (FJNU), Fujian Normal University, Fuzhou 350117, China; cbl990903@163.com (B.C.); dmli079@fjnu.edu.cn (D.L.); 2 Sino-French Institute of Nuclear Engineering and Technology, Sun Yat-sen University, Zhuhai 519082, China

**Keywords:** discrete Boltzmann method, Rayleigh–Taylor instability, initial inclined interface, compressible fluid, non-equilibrium effects

## Abstract

Rayleigh–Taylor (RT) instability is a basic fluid interface instability that widely exists in nature and in the engineering field. To investigate the impact of the initial inclined interface on compressible RT instability, the two-component discrete Boltzmann method is employed. Both the thermodynamic non-equilibrium (TNE) and hydrodynamic non-equilibrium (HNE) effects are studied. It can be found that the global average density gradient in the horizontal direction, the non-organized energy fluxes, the global average non-equilibrium intensity and the proportion of the non-equilibrium region first increase and then reduce with time. However, the global average density gradient in the vertical direction and the non-organized moment fluxes first descend, then rise, and finally descend. Furthermore, the global average density gradient, the typical TNE intensity and the proportion of non-equilibrium region increase with increasing angle of the initial inclined interface. Physically, there are three competitive mechanisms: (1) As the perturbed interface elongates, the contact area between the two fluids expands, which results in an increasing gradient of macroscopic physical quantities and leads to a strengthening of the TNE effects. (2) Under the influence of viscosity, the perturbation pressure waves on both sides of the material interface decrease with time, which makes the gradient of the macroscopic physical quantity decrease, resulting in a weakening of the TNE strength. (3) Due to dissipation and/or mutual penetration of the two fluids, the gradient of macroscopic physical quantities gradually diminishes, resulting in a decrease in the intensity of the TNE.

## 1. Introduction

The phenomenon of fluid interface instability caused by a less dense fluid supporting or accelerating a denser fluid in a gravitational field is called Rayleigh–Taylor (RT) instability [1,2]. As a fundamental hydrodynamic instability phenomenon, the RT instability is common in nature and engineering, such as inertial confinement fusion (ICF) [3,4,5], astrophysics [6,7,8], geophysics [9,10,11], atmospheric physics [12]. In the ICF, due to processing technology and other reasons, the surface of the target is not necessarily completely smooth, and there may be small defects, which will cause the uneven density of the target; at this time, the ablation surface and the acceleration are in a non-collinear state. Meanwhile, the non-collinear acceleration and density gradient will induce Kelvin–Helmholtz (KH) instability, making the RT process quite complicated [13]. Therefore, due to its great importance in practical engineering applications, it is necessary to study the RT instability under different perturbation interfaces.

In the past few decades, the RT instability under different disturbance interfaces has been extensively studied by scholars, and one of the configurations is that an unstable planar interface tilts away from the horizontal, i.e., inclined interface [13,14,15,16,17,18,19]. For example, Jiang et al., utilized high-speed shadowgraph technology to experimentally study the evolution of the interface induced by the RT instability at the inclined interface of immiscible fluids, and found that the influence of inclination effect on the width of mixing zone is mainly reflected in the later stage [13]. Andrews et al., studied the two-dimensional (2-D) mixing through the RT instability at a low-density ratio through a simple tilt experiment [14]. Liu et al., experimentally studied the mixing asymmetry in the turbulent mixing region of the RT instability at the inclined interface of immiscible fluids [15]. Holford et al., experimentally studied the mixing development of the RT instability at the inclined interface of two fluids under a gravity field, and analyzed the influence of inclination angle on fluid mixing efficiency. At the same time, the experimental results were verified using the compressible three-dimension numerical simulation method [16]. Youngs employed the direct numerical simulation method to solve the incompressible Navier–Stokes (NS) equations to study the effect of wall friction on the tilted RT instability [17]. Sahu et al., used the multiphase lattice Boltzmann method to simulate buoyancy-induced mixing in a tilted channel with a different Atwood number (At), Reynolds number (Re), inclination, and surface tension parameters [18]. Andrews et al., applied implicit large-eddy simulations and a direct numerical simulation technique to study the influence of wall effects on the tilted RT instability [19]. These studies help to enrich our in-depth understanding of the physical mechanism of interface instability.

Although many important results have been obtained during the numerical study of the RT instability with initial inclined interface, most of these numerical studies are based on hydrodynamic models at the macroscopic level, such as Euler or NS equations, focusing on the hydrodynamic non-equilibrium (HNE) effects during the RT process, while the thermodynamic non-equilibrium (TNE) effects are often ignored. In order to reveal the complex TNE effects in the process of compressible fluid flow, the discrete Boltzmann method (DBM) came into being [20,21,22,23,24]. The DBM is a coarse-grained physical model based on the non-equilibrium statistical physics, which is developed from the lattice Boltzmann method [25,26,27,28,29,30]. From the perspective of physical modeling, the DBM is approximately equivalent to a continuous fluid model plus a coarse-grained model describing TNE effects. In recent years, the DBM has been widely used in the numerical study of the RT instability in compressible fluids and has made remarkable progress [31,32,33,34,35,36,37,38,39,40]. For example, Lai et al., studied the impact of compressibility on the RT instability by using the DBM and found that the compressibility effect and the global TNE intensity exhibit opposite tendencies in the early and later stages of the RT instability [31]. Chen et al., adopted the DBM to numerically study the RT instability of multi-mode initial perturbations in compressible fluids [32]. Chen et al., studied the effects of interfacial tension, viscosity, and heat conduction on the 2-D compressible RT instability by using the DBM with the van der Waals equation of state [33]. Chen et al., used the DBM with multiple relaxation times to study the influence of the length of the morphological boundary and the TNE intensity on the complex configuration and dynamic process of the coupled Rayleigh–Taylor–Kelvin–Helmholtz instability system [34]. Zhang et al., studied the fine structure and various non-equilibrium behaviors of the RT instability flow near the interface in a miscible two-fluid system by using the compressible DBM with tracer, and discussed the effects of compressibility and viscosity on the RT instability mixing [35]. Chen et al., used the DBM to study the evolution of the compressible RT instability at several different specific heat ratios [36]. Ye et al., studied the effect of Knudsen number on the RT instability in 2-D compressible fluid using the DBM [37]. Li et al., simulated the nonlinear evolution of the multi-mode compressible RT instability using the DBM [38]. Chen et al., studied the impacts of viscosity, heat conduction, and Prandtl number on 2-D RT instability by using the multi-relaxation time DBM simulation with gravity [39]. Li et al., utilized the DBM with tracers to study the influence of viscosity, acceleration, compressibility, and At on the 2-D compressible RT instability under multi-mode perturbation [40]. These studies provide us with a series of new understandings of the rich TNE behaviors during the evolution of the RT instability.

In general, physical models can be divided into two categories: single-component models and multi-component models. In the single-component DBM, only a single distribution function is used to describe the fluid system. The *N*-component DBM uses *N* distribution functions to describe the fluid system, and each distribution function describes a fluid component. The single-component DBM [31,39] can only be used to simulate the RT instability in a special case where the heavy cold medium is supported by a light hot one, while the two-component DBM can be used to study the RT instability in more general cases where the two components have independent temperatures. In order to describe the fluid system composed of two different components more accurately, the two-component DBM is considered. In recent years, the two-component DBM has made some progress in combustion [41], fluid instability [42,43,44] and other non-equilibrium flows [45]. Here, we focus on and briefly introduce the research results of two-component DBM in fluid instability. In fact, Lin et al., utilized the two-component DBM to explore the influence of Re on global non-equilibrium behaviors and the growth rate of the entropy of mixing in the RT process [42]. Zhang et al., adopted a two-fluid DBM to investigate the effect of Prandtl number on KH instability [43]. Lin et al., used the two-component DBM to study the non-equilibrium effect in the evolution of 2-D KH instability [44].

Here, the effect of the inclination angle on the inclined interface compressible RT instability is numerically studied by means of the two-component DBM, and both HNE and TNE effects in the evolution of the system are studied. The remaining structure of this paper is as follows: In Section 2, the two-component DBM is introduced. In Section 3, the numerical simulation. The influence of initial inclined interface on compressible RT instability is simulated and analyzed. In Section 4, a succinct conclusion is provided.

## 2. Two-Component DBM

In this paper, the discrete Boltzmann equation of the system containing two components, σ=A and *B* with independent specific heat ratios, is as follows:

(1) $$\frac{\partial f_{i}^{\sigma }}{\partial t}+v_{i\alpha }^{\sigma }\frac{\partial f_{i}^{\sigma }}{\partial r_{\alpha }}=\Omega_{i}^{\sigma }+G_{i}^{\sigma },$$

where $f_{i}^{\sigma }$ is the discrete distribution function, $r_{\alpha }$ represents the Cartesian coordinate in the α direction, $v_{i\alpha }^{\sigma }$ denotes the discrete velocity, i=1,2,⋯,N. $\Omega_{i}^{\sigma }$ (the collision term) and $G_{i}^{\sigma }$ (the force term) decribe the change rates of distribution function under molecular collision and external force, respectively.

The collision term is first linearized as follows:

(2) $$\Omega_{i}^{\sigma }=-\frac{1}{\tau^{\sigma }}\left(f_{i}^{\sigma }-f_{i}^{\sigma eq}\right),$$

where $\tau^{\sigma }=1/\left(n^{A}/\theta^{A}+n^{B}/\theta^{B}\right)$ represents the relaxation time, it depends on the particle number density $n^{\sigma }$ and two flexible parameters $\left(\theta^{A},\theta^{B}\right)$. $f_{i}^{\sigma eq}$ is the discretization of the equilibrium distribution function $f^{\sigma eq}$:

(3) $$\begin{array}{c} f^{\sigma eq}=n^{\sigma }{\left(\frac{m^{\sigma }}{2\pi kT}\right)}^{D/2}{\left(\frac{m^{\sigma }}{2\pi I^{\sigma }kT}\right)}^{1/2}\exp\left[-\frac{m^{\sigma }{\left|v-u\right|}^{2}}{2kT}-\frac{m^{\sigma }\eta^{2}}{2I^{\sigma }kT}\right], \end{array}$$

where $m^{\sigma }$ is the particle mass, *T* is the mixture temperature, *D* is the number of the spatial dimension (here, D=2), and u is the mixture velocity and the Boltzmann constant k=1. $I^{\sigma }$ is the extra degree of freedom. v is the velocity of particle translational motion, and the internal energies in extra degrees of freedom corresponding to molecular rotation and/or vibration can be described by $\eta^{2}$.

The force term $G_{i}^{\sigma }$ is as follows:

(4) $$G_{i}^{\sigma }=\frac{m^{\sigma }a_{\alpha }}{T^{\sigma }}\left(v_{i\alpha }-u_{\alpha }^{\sigma }\right)f_{i}^{\sigma eq},$$

where $a_{\alpha }$ is the body acceleration in the α direction.

In order to construct the DBM at NS level, according to the Chapman–Enskog multi-scale analysis, the discrete equilibrium distribution function $f_{i}^{\sigma eq}$ needs to satisfy the following seven kinetic moments in the process of discretization of particle velocity:

(5) $$\;\int \int \;f^{\sigma eq}dvd\eta =\sum_{i}f_{i}^{\sigma eq}=n^{\sigma },$$

(6) $$\;\int \int \;f^{\sigma eq}v_{\alpha }dvd\eta =\sum_{i}f_{i}^{\sigma eq}v_{i\alpha }^{\sigma }=n^{\sigma }u_{\alpha },$$

(7) $$\;\int \int \;f^{\sigma eq}\left(v^{2}+\eta^{2}\right)dvd\eta =\sum_{i}f_{i}^{\sigma eq}\left(v_{i}^{\sigma 2}+\eta_{i}^{\sigma 2}\right)=n^{\sigma }\left[\left(D+I^{\sigma }\right)\frac{T}{m^{\sigma }}+u^{2}\right],$$

(8) $$\;\int \int \;f^{\sigma eq}v_{\alpha }v_{\beta }dvd\eta =\sum_{i}f_{i}^{\sigma eq}v_{i\alpha }^{\sigma }v_{i\beta }^{\sigma }=n^{\sigma }\left(\delta_{\alpha \beta }\frac{T}{m^{\sigma }}+u_{\alpha }u_{\beta }\right),$$

(9) $$\begin{array}{cc} \;\int \int \;f^{\sigma eq}\left(v^{2}+\eta^{2}\right)v_{\alpha }dvd\eta = & \sum_{i}f_{i}^{\sigma eq}\left(v_{i}^{\sigma 2}+\eta_{i}^{\sigma 2}\right)v_{i\alpha }^{\sigma }=n^{\sigma }u_{\alpha }\left[\left(D+I^{\sigma }+2\right)\frac{T}{m^{\sigma }}+u^{2}\right], \end{array}$$

(10) $$\begin{array}{cc} \;\int \int \;f^{\sigma eq}v_{\alpha }v_{\beta }v_{\chi }dvd\eta = & \sum_{i}f_{i}^{\sigma eq}v_{i\alpha }^{\sigma }v_{i\beta }^{\sigma }v_{i\chi }^{\sigma }=n^{\sigma }\left(u_{\alpha }\delta_{\beta \chi }+u_{\beta }\delta_{\alpha \chi }+u_{\chi }\delta_{\alpha \beta }\right)\frac{T}{m^{\sigma }}+n^{\sigma }u_{\alpha }u_{\beta }u_{\chi }, \end{array}$$

(11) $$\begin{array}{cc} \;\int \int \;f^{\sigma eq}\left(v^{2}+\eta^{2}\right)v_{\alpha }v_{\beta }dvd\eta & =\sum_{i}f_{i}^{\sigma eq}\left(v_{i}^{\sigma 2}+\eta_{i}^{\sigma 2}\right)v_{i\alpha }^{\sigma }v_{i\beta }^{\sigma }=n^{\sigma }\delta_{\alpha \beta }\left[\left(D+I^{\sigma }+2\right)\frac{T}{m^{\sigma }}+u^{2}\right]\frac{T}{m^{\sigma }}\\ \; & +n^{\sigma }u_{\alpha }u_{\beta }\left[\left(D+I^{\sigma }+2\right)\frac{T}{m^{\sigma }}+u^{2}+\frac{2T}{m^{\sigma }}\right], \end{array}$$

where $\delta_{\alpha \beta }$ denotes the Kronecker function, and α, β, χ = *x* or *y*.

In fact, the above seven kinetic moments can be expressed in the following matrix form:

(12) $$M\;\cdot f^{\sigma eq}={\hat{f}}^{\sigma eq},$$

where

(13) $$M={\left(M_{1},M_{2},\cdots ,M_{N}\right)}^{T},$$

(14) $$f^{\sigma eq}={\left(f_{1}^{\sigma eq},f_{2}^{\sigma eq},\cdots ,f_{N}^{\sigma eq}\right)}^{T},$$

(15) $${\hat{f}}^{\sigma eq}={\left({\hat{f_{1}}}^{\sigma eq},{\hat{f_{2}}}^{\sigma eq},\cdots ,{\hat{f_{N}}}^{\sigma eq}\right)}^{T},$$

$M_{i}={\left(m_{i1},m_{i2},\cdots ,m_{iN}\right)}^{T}$, to be specific,

$$m_{i1}=1,m_{i2}=v_{ix}^{\sigma },m_{i3}=v_{iy}^{\sigma },m_{i4}=v_{ix}^{\sigma 2}+v_{iy}^{\sigma 2}+\eta_{i}^{\sigma 2},m_{i5}=v_{ix}^{\sigma }v_{iy}^{\sigma },$$

$$m_{i6}=v_{ix}^{\sigma 2},m_{i7}=v_{iy}^{\sigma 2},m_{i8}=v_{ix}^{\sigma }\left(v_{ix}^{\sigma 2}+v_{iy}^{\sigma 2}+\eta_{i}^{\sigma 2}\right),m_{i9}=v_{iy}^{\sigma }\left(v_{ix}^{\sigma 2}+v_{iy}^{\sigma 2}+\eta_{i}^{\sigma 2}\right),$$

$$m_{i10}=v_{ix}^{\sigma 3},m_{i11}=v_{iy}^{\sigma 3},m_{i12}=v_{ix}^{\sigma 2}v_{iy}^{\sigma },m_{i13}=v_{ix}^{\sigma }v_{iy}^{\sigma 2},$$

$$m_{i14}=v_{ix}^{\sigma }v_{iy}^{\sigma }\left(v_{ix}^{\sigma 2}+v_{iy}^{\sigma 2}+\eta_{i}^{\sigma 2}\right),m_{i15}={v_{ix}}^{\sigma 2}\left(v_{ix}^{\sigma 2}+v_{iy}^{\sigma 2}+\eta_{i}^{\sigma 2}\right),m_{i16}={v_{iy}}^{\sigma 2}\left(v_{ix}^{\sigma 2}+v_{iy}^{\sigma 2}+\eta_{i}^{\sigma 2}\right).$$

The elements in the moment ${\hat{f}}^{\sigma eq}$ are expressed as

$${\hat{f_{1}}}^{\sigma eq}=\rho^{\sigma },{\hat{f_{2}}}^{\sigma eq}=\rho^{\sigma }u_{x},{\hat{f_{3}}}^{\sigma eq}=\rho^{\sigma }u_{y},{\hat{f_{4}}}^{\sigma eq}=\rho^{\sigma }\left[\left(D+I^{\sigma }\right)T+u^{2}\right],$$

$${\hat{f_{5}}}^{\sigma eq}=\rho^{\sigma }\left(T+u_{x}^{2}\right),{\hat{f_{6}}}^{\sigma eq}=\rho^{\sigma }u_{x}u_{y},{\hat{f_{7}}}^{\sigma eq}=\rho^{\sigma }\left(T+u_{y}^{2}\right),$$

$${\hat{f_{8}}}^{\sigma eq}=\rho^{\sigma }u_{x}\left[\left(D+I^{\sigma }+2\right)T+u^{2}\right],{\hat{f_{9}}}^{\sigma eq}=\rho^{\sigma }u_{y}\left[\left(D+I^{\sigma }+2\right)T+u^{2}\right],$$

$${\hat{f_{10}}}^{\sigma eq}=3\rho^{\sigma }u_{x}T+\rho^{\sigma }u_{x}^{3},{\hat{f_{11}}}^{\sigma eq}=\rho^{\sigma }u_{y}T+\rho^{\sigma }u_{x}^{2}u_{y},$$

$${\hat{f_{12}}}^{\sigma eq}=\rho^{\sigma }u_{x}T+\rho^{\sigma }u_{x}u_{y}^{2},{\hat{f_{13}}}^{\sigma eq}=3\rho^{\sigma }u_{y}T+\rho^{\sigma }u_{y}^{3},$$

$${\hat{f_{14}}}^{\sigma eq}=\rho^{\sigma }T\left[\left(D+I^{\sigma }+2\right)T+u^{2}\right]+\rho^{\sigma }u_{x}^{2}\left[\left(D+I^{\sigma }+4\right)T+u^{2}\right],$$

$${\hat{f_{15}}}^{\sigma eq}=\rho^{\sigma }u_{x}u_{y}\left[\left(D+I^{\sigma }+4\right)T+u^{2}\right],$$

$${\hat{f_{16}}}^{\sigma eq}=\rho^{\sigma }T\left[\left(D+I^{\sigma }+2\right)T+u^{2}\right]+\rho^{\sigma }u_{y}^{2}\left[\left(D+I^{\sigma }+4\right)T+u^{2}\right],$$

where $\rho^{\sigma }$ represents the mass density.

Under the premise of the existence of $M^{-1}$, from Equation (12), the discrete equilibrium distribution function can be obtained as follows:

(16) $$f^{\sigma eq}=M^{-1}\;\cdot \;{\hat{f}}^{\sigma eq}.$$

**Figure 1:**
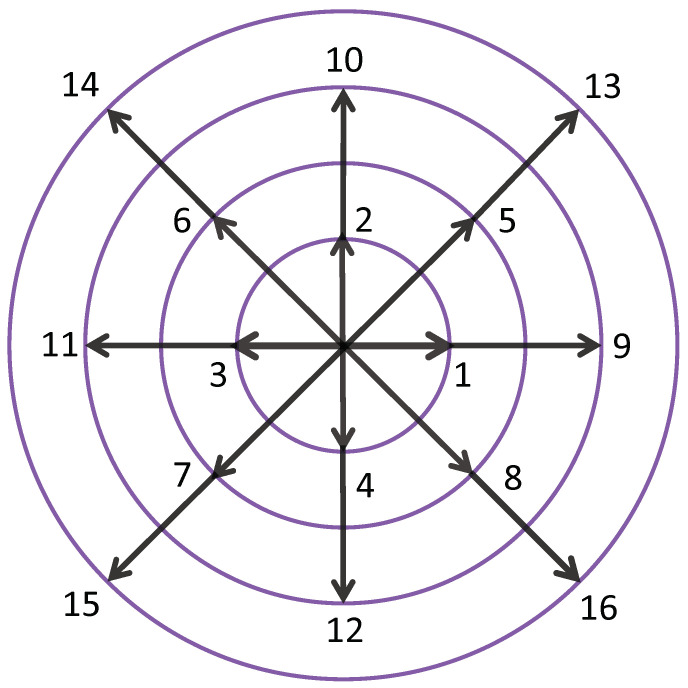
D2V16 discrete velocity model diagram.

The discrete equilibrium distribution function should be expressed explicitly, and this is a crucial step in constructing the DBM. In order to obtain the discrete equilibrium distribution function, it is necessary to ensure that the number of discrete velocities in the discrete velocity model is not less than the number of kinetic moments needed. Since the kinetic moment relationship in Equation (12) is used to recover the compressible NS equations, and Equation (12) contains 16 independent variables, the D2V16 discrete velocity model is adopted in this paper, as shown in Figure 1. The mathematical expression is as follows:

(17) $$v_{i}=\left\{\begin{array}{c} v_{a}\left[\cos\frac{(i-1)\pi }{2},\sin\frac{(i-1)\pi }{2}\right],i=1,\cdots ,4,\\ v_{b}\left[\cos\frac{(2i-1)\pi }{4},\sin\frac{(2i-1)\pi }{4}\right],i=5,\cdots ,8,\\ v_{c}\left[\cos\frac{(i-9)\pi }{2},\sin\frac{(i-9)\pi }{2}\right],i=9,\cdots ,12,\\ v_{d}\left[\cos\frac{(2i-9)\pi }{4},\sin\frac{(2i-9)\pi }{4}\right],i=13,\cdots ,16, \end{array}\right.$$

besides, $\eta_{i}=\eta_{0}$, when i=5,⋯,8; otherwise, $\eta_{i}=0$.

Using the Chapman–Enskog multi-scale analysis, the density distribution function, time derivative, space derivative and external force term can be expanded as follows:

(18) $$\left\{\begin{array}{c} f_{i}^{\sigma }=f_{i}^{\sigma (0)}+\varepsilon f_{i}^{\sigma (1)}+\varepsilon^{2}f_{i}^{\sigma (2)}+\cdots ,\\ \frac{\partial }{\partial t}=\varepsilon \frac{\partial }{\partial t_{1}}+\varepsilon^{2}\frac{\partial }{\partial t_{2}}+\cdots ,\\ \frac{\partial }{\partial r_{\alpha }}=\varepsilon \frac{\partial }{\partial r_{1\alpha }},\\ a_{\alpha }=\varepsilon a_{1\alpha }, \end{array}\right.$$

it should be noted that when restoring the macroscopic fluid mechanics equation at the level of NS equation, the time scale expansion only needs to use $\frac{\partial }{\partial t}\approx \varepsilon \frac{\partial }{\partial t_{1}}+\varepsilon^{2}\frac{\partial }{\partial t_{2}}$, and the macroscopic fluid mechanics equation at the level of NS equation can be obtained:

(19) $$\frac{\partial \rho^{\sigma }}{\partial t}+\frac{\partial }{\partial r_{\alpha }}\left(\rho^{\sigma }u_{\alpha }^{\sigma }\right)=0,$$

(20) $$\frac{\partial }{\partial t}\left(\rho^{\sigma }u_{\alpha }^{\sigma }\right)+\frac{\partial }{\partial r_{\beta }}\left(\delta_{\alpha \beta }p^{\sigma }+\rho^{\sigma }u_{\alpha }^{\sigma }u_{\beta }^{\sigma }\right)+\frac{\partial }{\partial r_{\beta }}\left(P_{\alpha \beta }^{\sigma }+U_{\alpha \beta }^{\sigma }\right)=\rho^{\sigma }a_{\alpha }-\frac{\rho^{\sigma }}{\tau^{\sigma }}\left(u_{\alpha }^{\sigma }-u_{\alpha }\right),$$

(21) $$\begin{array}{cc} \; & \frac{\partial }{\partial t}\;\rho^{\sigma }\left(e^{\sigma }+\frac{1}{2}u^{\sigma 2}\right)+\frac{\partial }{\partial r_{\alpha }}\left[\rho^{\sigma }u_{\alpha }^{\sigma }\left(e^{\sigma }+\frac{1}{2}u^{\sigma 2}\right)+p^{\sigma }u_{\alpha }^{\sigma }\right]\\ & -\frac{\partial }{\partial r_{\alpha }}\left[\kappa^{\sigma }\frac{\partial }{\partial r_{\alpha }}\left(\frac{D+I^{\sigma }}{2}\frac{T^{\sigma }}{m^{\sigma }}\right)-u_{\beta }^{\sigma }P_{\alpha \beta }^{\sigma }+X_{\alpha }^{\sigma }\right]\\ & =\rho^{\sigma }u_{\alpha }^{\sigma }a_{\alpha }-\frac{\rho^{\sigma }}{\tau^{\sigma }}\left(\frac{D+I^{\sigma }}{2}\frac{T^{\sigma }-T}{m^{\sigma }}+\frac{u^{\sigma 2}-u^{2}}{2}\right), \end{array}$$

with

(22) $$\begin{array}{c} P_{\alpha \beta }^{\sigma }=-\mu^{\sigma }\left(\frac{\partial u_{\alpha }^{\sigma }}{\partial r_{\beta }}+\frac{\partial u_{\beta }^{\sigma }}{\partial r_{\alpha }}-\frac{2\delta_{\alpha \beta }}{D+I^{\sigma }}\frac{\partial u_{\chi }^{\sigma }}{\partial r_{\chi }}\right), \end{array}$$

(23) $$\begin{array}{c} U_{\alpha \beta }^{\sigma }=-\rho^{\sigma }\left(\delta_{\alpha \beta }\frac{u^{\sigma 2}+u^{2}-2u_{\chi }^{\sigma }u_{\chi }}{D+I^{\sigma }}+u_{\alpha }u_{\beta }^{\sigma }+u_{\alpha }^{\sigma }u_{\beta }-u_{\alpha }^{\sigma }u_{\beta }^{\sigma }-u_{\alpha }u_{\beta }\right), \end{array}$$

(24) $$\begin{array}{c} X_{\alpha }^{\sigma }=\frac{\rho^{\sigma }u_{\alpha }^{\sigma }}{D+I^{\sigma }}{\left(u_{\beta }^{\sigma }-u_{\beta }\right)}^{2}-\rho^{\sigma }\frac{u_{\alpha }^{\sigma }-u_{\alpha }}{2}\left[\frac{D+I^{\sigma }+2}{m^{\sigma }}\left(T^{\sigma }-T\right)+u^{\sigma 2}-u^{2}\right], \end{array}$$

where $p^{\sigma }=n^{\sigma }T^{\sigma }$ stands for the pressure, $\mu^{\sigma }=p^{\sigma }\tau^{\sigma }$ represents the dynamic viscosity coefficient, and $\kappa^{\sigma }=\left[\left(D+I^{\sigma }+2\right)/\left(D+I^{\sigma }\right)\right]\mu^{\sigma }$ denotes the heat conductivity.

Using the operator $\sum_{\sigma }$ on both sides of Equations (19)–(21) leads to the NS equations describing the whole system,

(25) $$\frac{\partial \rho }{\partial t}+\frac{\partial }{\partial r_{\alpha }}\left(\rho u_{\alpha }\right)=0,$$

(26) $$\frac{\partial }{\partial t}\left(\rho u_{\alpha }\right)+\frac{\partial }{\partial r_{\beta }}\sum_{\sigma }\left(\delta_{\alpha \beta }P^{\sigma }+\rho^{\sigma }u_{\alpha }^{\sigma }u_{\beta }^{\sigma }\right)+\frac{\partial }{\partial r_{\beta }}\sum_{\sigma }P_{\alpha \beta }^{\sigma }+U_{\alpha \beta }^{\sigma }=\rho a_{\alpha },$$

(27) $$\begin{array}{c} \begin{array}{cc} & \frac{\partial }{\partial t}\left[\rho (e+\frac{1}{2}u^{2})\right]+\frac{\partial }{\partial r_{\alpha }}\sum_{\sigma }\left[\rho^{\sigma }u_{\alpha }^{\sigma }\left(e^{\sigma }+\frac{1}{2}u^{\sigma 2}\right)+p^{\sigma }u_{\alpha }^{\sigma }\right]\\ & -\frac{\partial }{\partial r_{\alpha }}\sum_{\sigma }\left[k^{\sigma }\frac{\partial }{\partial r_{\alpha }}\left(\frac{D+I^{\sigma }}{2}\frac{T^{\sigma }}{m^{\sigma }}\right)-u_{\beta }^{\sigma }P_{\alpha \beta }^{\sigma }+X_{\alpha }^{\sigma }\right]=\rho u_{\alpha }a_{\alpha },\; \end{array} \end{array}$$

where $e=\sum_{\sigma }\rho^{\sigma }\left(e^{\sigma }+u^{\sigma 2}/2\right)/\rho -u^{2}/2$ denotes the internal energy of the whole system per unit mass.

Among the above seven kinetic moment relations, Equations (5)–(7) satisfy the conservation of mass, momentum and energy, respectively. Therefore, $f^{\sigma eq}$ (and ${f_{i}}^{\sigma eq}$) can be replaced by $f^{\sigma }$ (and ${f_{i}}^{\sigma }$) in these three formulas. However, for the four moment relations (8)–(11), the values on the left and right sides of the formula may deviate if the same replacement is performed. According to the non-equilibrium statistical physics, this deviation can be used to describe the TNE of the system. To be specific, the following non-equilibrium effect quantities are introduced to describe the degree of deviation of the system from the thermodynamic equilibrium:

(28) $$\Delta_{m,n}^{\sigma *}=m^{\sigma }\left[M_{m,n}^{*}\left({f_{i}}^{\sigma }\right)-M_{m,n}^{*}\left({f_{i}}^{\sigma eq}\right)\right],$$

where

(29) $$\left\{\begin{array}{c} M_{2}^{*}\left(f_{i}^{\sigma }\right)=\sum_{i}{f_{i}}^{\sigma }\left(v_{i}^{\sigma }-u\right)\left(v_{i}^{\sigma }-u\right),\\ M_{3}^{*}\left(f_{i}^{\sigma }\right)=\sum_{i}{f_{i}}^{\sigma }\left(v_{i}^{\sigma }-u\right)\;\cdot \;\left(v_{i}^{\sigma }-u\right)\left(v_{i}^{\sigma }-u\right),\\ M_{3,1}^{*}\left(f_{i}^{\sigma }\right)=\sum_{i}{f_{i}}^{\sigma }\left[\left(v_{i}^{\sigma }-u\right)\;\cdot \;\left(v_{i}^{\sigma }-u\right)+\eta_{i}^{\sigma 2}\right]\left(v_{i}^{\sigma }-u\right),\\ M_{4,2}^{*}\left(f_{i}^{\sigma }\right)=\sum_{i}{f_{i}}^{\sigma }\left[\left(v_{i}^{\sigma }-u\right)\;\cdot \;\left(v_{i}^{\sigma }-u\right)+\eta_{i}^{\sigma 2}\right]\left(v_{i}^{\sigma }-u\right)\left(v_{i}^{\sigma }-u\right), \end{array}\right.$$

(30) $$\left\{\begin{array}{c} M_{2}^{*}\left(f_{i}^{\sigma eq}\right)=\sum_{i}{f_{i}}^{\sigma eq}\left(v_{i}^{\sigma }-u\right)\left(v_{i}^{\sigma }-u\right),\\ M_{3}^{*}\left(f_{i}^{\sigma eq}\right)=\sum_{i}{f_{i}}^{\sigma eq}\left(v_{i}^{\sigma }-u\right)\;\cdot \;\left(v_{i}^{\sigma }-u\right)\left(v_{i}^{\sigma }-u\right),\\ M_{3,1}^{*}\left(f_{i}^{\sigma eq}\right)=\sum_{i}{f_{i}}^{\sigma eq}\left[\left(v_{i}^{\sigma }-u\right)\;\cdot \;\left(v_{i}^{\sigma }-u\right)+\eta_{i}^{\sigma 2}\right]\left(v_{i}^{\sigma }-u\right),\\ M_{4,2}^{*}\left(f_{i}^{\sigma eq}\right)=\sum_{i}{f_{i}}^{\sigma eq}\left[\left(v_{i}^{\sigma }-u\right)\;\cdot \;\left(v_{i}^{\sigma }-u\right)+\eta_{i}^{\sigma 2}\right]\left(v_{i}^{\sigma }-u\right)\left(v_{i}^{\sigma }-u\right), \end{array}\right.$$

$M_{m,n}^{*}$ is the central moment; the subscript “m,n” denoting the *m*-order tensor is reduced to the *n*-order tensor.

In order to describe the global TNE effects of the fluid system more specifically, the following non-equilibrium quantities are defined:

(31) $$|\Delta_{2}^{\sigma *}|=|\Delta_{2xx}^{\sigma *}|+|\Delta_{2xy}^{\sigma *}|+|\Delta_{2yy}^{\sigma *}|,$$

(32) $$|\Delta_{3,1}^{\sigma *}|=|\Delta_{3,1x}^{\sigma *}|+|\Delta_{3,1y}^{\sigma *}|,$$

(33) $$|\Delta_{3}^{\sigma *}|=|\Delta_{3xxx}^{\sigma *}|+|\Delta_{3xxy}^{\sigma *}|+|\Delta_{3xyy}^{\sigma *}|+|\Delta_{3yyy}^{\sigma *}|,$$

(34) $$|\Delta_{4,2}^{\sigma *}|=|\Delta_{4,2xx}^{\sigma *}|+|\Delta_{4,2xy}^{\sigma *}|+|\Delta_{4,2yy}^{\sigma *}|.$$

Physically, $\Delta_{2}^{\sigma *}$ stands for the non-organized momentum and is related to viscosity, $\Delta_{3,1}^{\sigma *}$ and $\Delta_{3}^{\sigma *}$ denote the non-organized energy flux and are related to heat flux, and $\Delta_{4,2}^{\sigma *}$ signifies the flux of non-organized energy flux.

By summing up several non-equilibrium quantities defined above, the global TNE quantity can be obtained:

(35) $$|\Delta^{\sigma *}|=|\Delta_{2}^{\sigma *}|+|\Delta_{3,1}^{\sigma *}|+|\Delta_{4,2}^{\sigma *}|+|\Delta_{3}^{\sigma *}|,$$

which can describe the degree of deviation from the equilibrium state as a whole.

Based on the above global TNE quantities, the global average TNE strength is further defined as:

(36) $${\bar{D}}^{\sigma }=\frac{1}{L_{x}L_{y}}\int_{0}^{L_{x}}\int_{0}^{L_{y}}\left|\Delta^{\sigma *}\right|dxdy,$$

the global average viscous stress tensor strength:

(37) $${\bar{D}}_{2}^{\sigma }=\frac{1}{L_{x}L_{y}}\int_{0}^{L_{x}}\int_{0}^{L_{y}}\left|\Delta_{2}^{\sigma *}\right|dxdy,$$

and the global average heat flux strength:

(38) $${\bar{D}}_{3,1}^{\sigma }=\frac{1}{L_{x}L_{y}}\int_{0}^{L_{x}}\int_{0}^{L_{y}}\left|\Delta_{3,1}^{\sigma *}\right|dxdy,$$

where $L_{x}$ and $L_{y}$ represent the length and width of the computational domain, respectively.

## 3. Numerical Simulations and Discussion

First of all, the numerical validation is performed in Appendix A. Then, the system of the compressible RT instability with the constant gravity acceleration a=(0,−g) is simulated. Due to the influence of the gravity field, the pressure in the system increases from top to bottom. The initial flow field satisfies the static equilibrium condition:

(39) ∇p=ρa.

**Figure 2:**
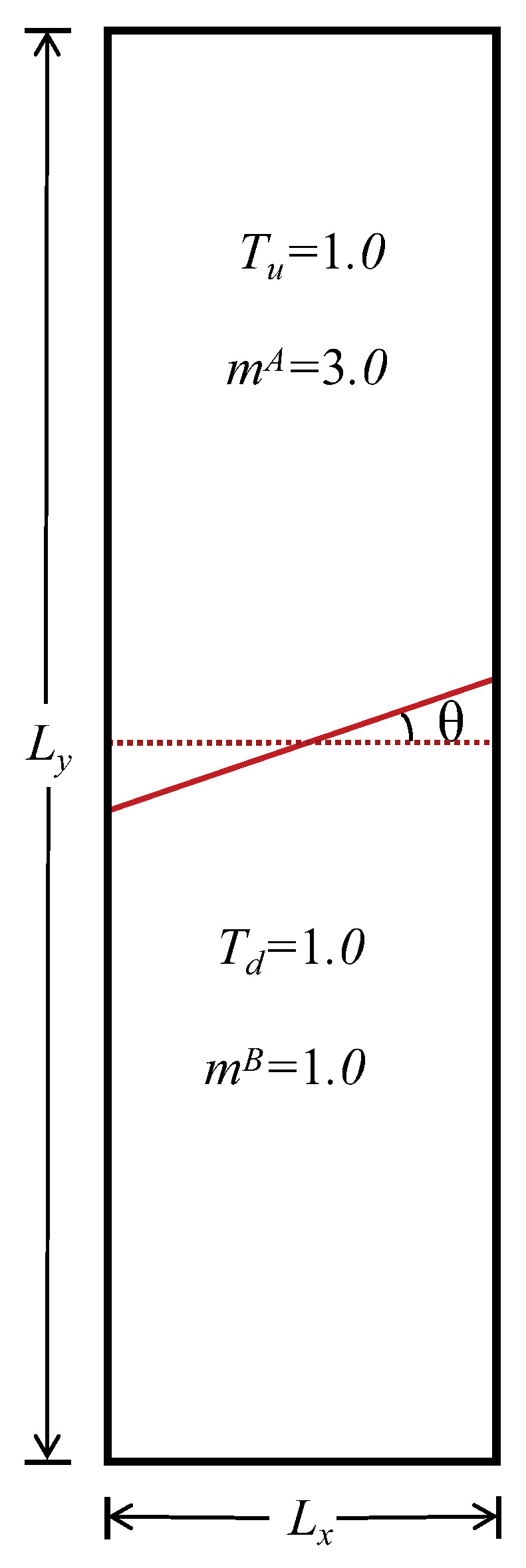
The initial configuration of the compressible RT instability.

The initial instability conditions of the fluid system are adopted as:

(40) $$\begin{array}{c} \left\{\begin{array}{c} T(x,y)=T_{u},\;n^{A}=\frac{p_{m}}{T_{u}}\exp\left[\frac{m^{A}g}{T_{u}}\left(y_{m}\left(x\right)-y\right)\right],\;\\ n^{B}=0,\;y>y_{m}\left(x\right),\\ T(x,y)=T_{d},\;n^{B}=\frac{p_{m}}{T_{d}}\exp\left[\frac{m^{B}g}{T_{d}}\left(y_{m}\left(x\right)-y\right)\right],\;\\ n^{A}=0,\;y<y_{m}\left(x\right), \end{array}\right. \end{array}$$

where the initial disturbance of the interface $y_{m}\left(x\right)$ satisfies [46]

(41) $$y_{m}\left(x\right)=\frac{L_{y}-L_{x}\tan\theta }{2}+x\tan\theta ,$$

where the subscript *m* represents the material interface, θ denotes the inclination angle of the initial interface. and $T_{u}$ and $T_{d}$ represent the initial temperatures of the upper and lower parts of the physical system. Considering that the physical quantity near the fluid interface is smooth in the actual physical system, the strong discontinuity of the physical field near the interface is smoothed by the hyperbolic tangent function tanh. Thus, the initial temperature field is

(42) $$T(x,y)=\frac{T_{u}+T_{d}}{2}+\frac{T_{u}-T_{d}}{2}\tanh\frac{y-y_{m}\left(x\right)}{W},$$

where $W=L_{y}/200$ denotes the width of the interfacial transition layer (here, $L_{y}=0.2$). In addition, the mirror–reflection boundary conditions are used in all directions. The initial configuration of the compressible RT instability is shown in Figure 2.

The grid convergence analysis is first performed to verify the resolution in Appendix B. In the numerical simulation, the computational grid is $N_{x}\times N_{y}=200\times 1600$, the corresponding spatial step $\Delta x=\Delta y=1.25\times 10^{-4}$, the time step $\Delta t=2.5\times 10^{-6}$, the material interface pressure $p_{m}=4.0$, and the relaxation parameter $\tau =4.0\times 10^{-5}$. The initial temperatures of the upper and lower parts of the fluid system are $T_{u}=T_{d}=1.0$. And the other parameters are $\left(v_{a},v_{b},v_{c},v_{d}\right)=\left(4.2,2.3,0.3,0.5\right)$, $\eta_{0}=5.3$, $m_{A}=3.0$, $m_{B}=1.0$, $a_{x}=0.0$, $a_{y}=-g=-2.0$.

**Figure 3:**
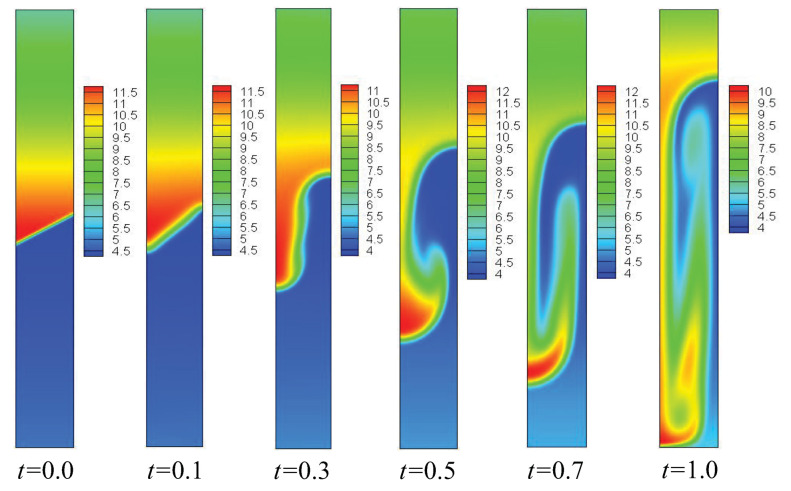
Contours of density in the case of θ=30° at times *t* = 0.0, 0.1, 0.3, 0.5, 0.7, and 1.0, respectively.

**Figure 4:**
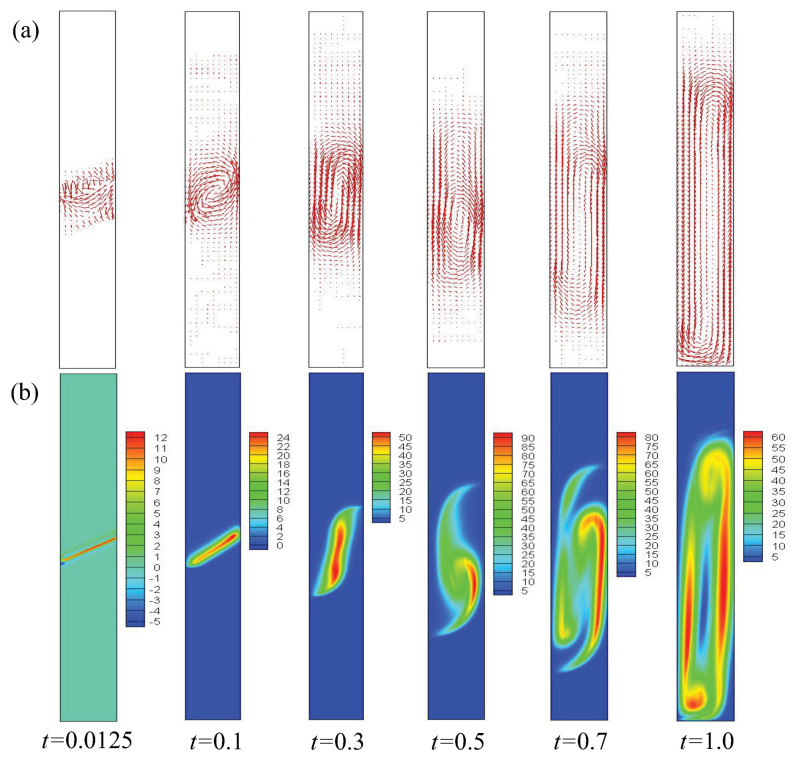
Snapshots of velocity and vorticity field of the RT system in the case of θ=30° at times *t* = 0.0125, 0.1, 0.3, 0.5, 0.7, and 1.0, respectively. The first and second rows are for the velocity (**a**) and vorticity (**b**), respectively.

To investigate the effect of the initial inclined interface angle θ on the compressible RT instability, 5°, 10°, 15°, 20°, 25°, 30°, 35°, 40°, and 45° are chosen. In order to obtain a clear understanding of the evolution of the compressible RT instability, Figure 3 presents the contours of density in the case of θ=30° at six different time instants. It can be observed that, at the beginning, the discontinuous initial density interface is smoothed by material diffusion, and the transition layer widens at t=0.1. Subsequently, the fluid interface bends significantly and evolves into the shapes of “bubble” and “spike” at t=0.3. After that, under the effect of shear force, a small vortex structure appears near the spike. In the later stage (about t>0.5), the vortex develops further and the fluid interface becomes longer. Moreover, to further understand the structural changes in fluid flow and to obtain a clearer understanding of the dynamics of RT instability evolution, the velocity and the quantity of vorticity $w(=\partial_{x}u_{y}-\partial_{y}u_{x})$ are presented with θ=30°, as shown in Figure 4.

Firstly, the global average density gradients in fluid system are discussed. The formulas of the global average density gradients are

(43) $$|\bar{\nabla_{x}\rho }|=\int_{0}^{L_{x}}\int_{0}^{L_{y}}|\nabla_{x}\rho |dxdy/\left(L_{x}L_{y}\right),$$

(44) $$|\bar{\nabla_{y}\rho }|=\int_{0}^{L_{x}}\int_{0}^{L_{y}}|\nabla_{y}\rho |dxdy/\left(L_{x}L_{y}\right),$$

(45) $$|\bar{\nabla \rho }|=\int_{0}^{L_{x}}\int_{0}^{L_{y}}\left|\nabla \rho \right|dxdy/\left(L_{x}L_{y}\right).$$

**Figure 5:**
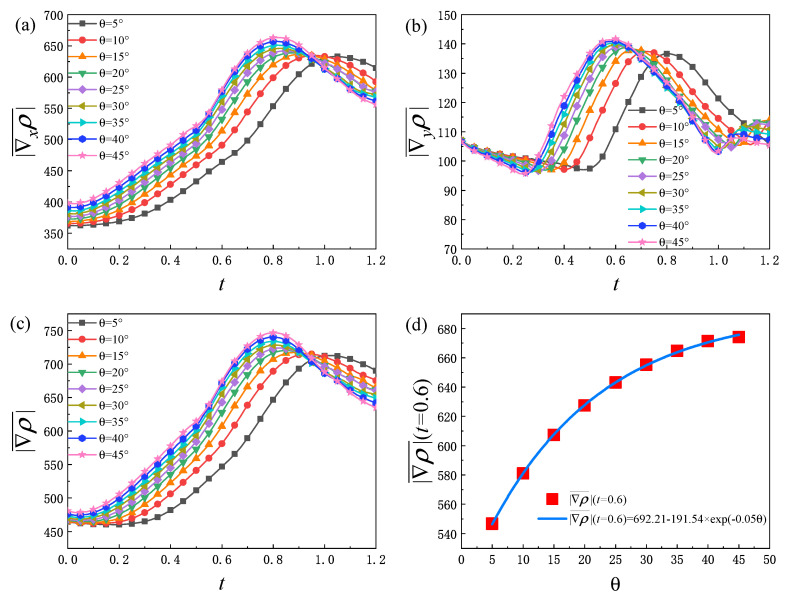
Evolution of global average density gradient with various initial interface inclination angles: (**a**) global average density gradient in *x* direction, (**b**) global average density gradient in *y* direction, (**c**) global average density gradient, (**d**) the functional relationship between the density gradient value and the initial interface inclination angles at t=0.6.

Figure 5a describes the evolution of the global average density gradient in *x* direction $|\bar{\nabla_{x}\rho }|$ with different θ. It can be found that the global average density gradient in the *x* direction increases with the increase in θ. And for each θ, the change trend of $|\bar{\nabla_{x}\rho }|$ increases first and then decreases. At the initial stage, $|\bar{\nabla_{x}\rho }|$ increases slightly. The physical reason for this phenomenon is that, during this period, the fluid system evolves slowly and the width of the transition layer grows slowly. Then, $|\bar{\nabla_{x}\rho }|$ increases rapidly. This is because, with the evolution of RT instability, the vortex structures on both sides of the interface begin to form. The overturning of the vortex structures further promote the interface elongation, the contact area of *A* and *B* components increases, and the vortex morphology becomes more complex. Besides, the mixing degree of *A* and *B* components in the fluid system is further deepened, and the local physical quantity gradient decreases. At this time, the tensile effect of the interface plays a leading role. Therefore, the global average density gradient in *x* direction increases rapidly. In the descending stage, the fluid interface continues to be elongated, but the mixing degree of the two components further deepens, the local physical quantity gradient decreases and the small vortex structure gradually disappears due to dissipation. Therefore, the global average density gradient in the *x* direction decreases.

Figure 5b is the evolution graph of the global average density gradient in the *y* direction $|\bar{\nabla_{y}\rho }|$ under different θ. It can be found that $|\bar{\nabla_{y}\rho }|$ decreases first, then increases, and finally decreases before the heavy medium reaches the bottom. In the process of evolution, there are three main physical mechanisms: (1) The disturbance wave gradient will decrease with time. (2) There exists a density difference on both sides of the intermediate material interface. Over time, the local density gradient decreases, and the gradient near the disturbance interface also decreases. (3) The perturbation interface is elongated and deformed, which leads to an increase in the gradient in the *y* direction. The above three mechanisms compete with each other and affect the development of $|\bar{\nabla_{y}\rho }|$ together. Take the case with θ=30° as an example: in the initial stage (about 0.0<t<0.25), the first mechanism plays a leading role, and the second mechanism and the third mechanism almost offset each other, so the $|\bar{\nabla_{y}\rho }|$ decreases. In the rising period (about 0.25<t<0.6), the third mechanism plays a leading role. In the declining phase (about t>0.6), the first mechanism and the second mechanism play a dominant role. In addition, as displayed in Figure 5c, the global average density gradient $|\bar{\nabla \rho }|$ first decreases, then increases, and finally decreases. In fact, the trend of $|\bar{\nabla \rho }|$ can be obtained from the analysis of $|\bar{\nabla_{x}\rho }|$ and $|\bar{\nabla_{y}\rho }|$ in Figure 5a,b. Furthermore, from Figure 5d, it can be found that there is an exponential relationship between the value of $|\bar{\nabla \rho }|$ at t=0.6 and the initial interface inclination angle in the rising period: $|\bar{\nabla \rho }|\left(t=0.6\right)=692.21-191.54\times \exp\left(-0.05\theta \right)$.

**Figure 6:**
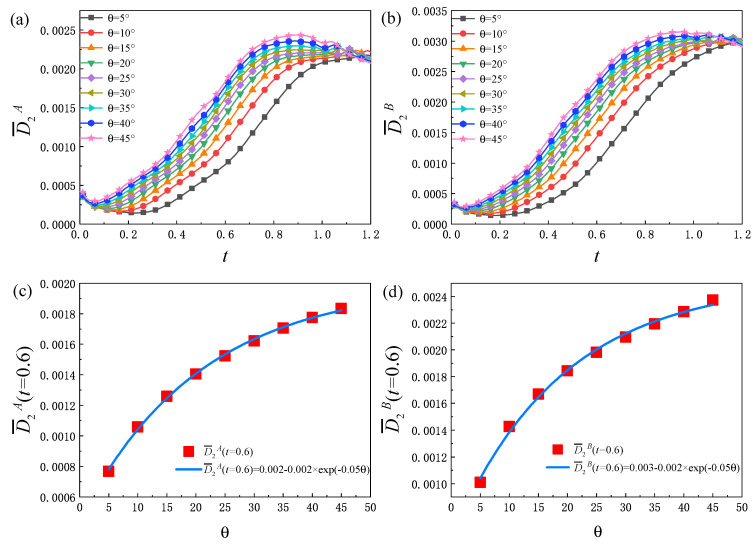
Evolution of the global average viscous stress tensor strength ${\bar{D}}_{2}^{\sigma }$ of components *A* (**a**) and *B* (**b**) under different initial interface inclination angles, the fitting curve of the value of ${\bar{D}}_{2}^{A}$ (**c**) and ${\bar{D}}_{2}^{B}$ (**d**) at t=0.6 and θ as: ${\bar{D}}_{2}^{A}\left(t=0.6\right)=0.002-0.002\times \exp\left(-0.05\theta \right)$ and ${\bar{D}}_{2}^{B}\left(t=0.6\right)=0.003-0.002\times \exp(-0.05\theta$), respectively.

Figure 6a,b plot the evolution of the non-equilibrium quantities ${\bar{D}}_{2}^{A}$ and ${\bar{D}}_{2}^{B}$, respectively. For different θ, ${\bar{D}}_{2}^{\sigma }$ first decreases, then increases, and finally decreases. At the beginning, the disturbance wave emerges around the material interface because the initial configuration is not set in a natural way, although there is a hyperbolic tangent function controlling the width of the smooth interface. Then, the wave departs from the material interface and propagates to both sides. During this process, the strength of the disturbance wave reduces and physical gradients near the disturbance wave decrease. Therefore, there is a decreasing tendency of ${\bar{D}}_{2}^{\sigma }$, which is related to the gradient of flow velocity. Afterwards, with the evolution of the RT instability, the non-uniformity of the flow velocity inside the system increases, so that ${\bar{D}}_{2}^{\sigma }$ increases. In the later stage, the spatial gradient of the flow velocity inside the system decreases, and ${\bar{D}}_{2}^{\sigma }$ shows a downward trend. Furthermore, ${\bar{D}}_{2}^{\sigma }$ increases with the increase in θ. This is because for a larger θ, the RT system evolves faster and involves more shear flows, which are related to the viscous shear tensor. In Figure 6c,d, the value of ${\bar{D}}_{2}^{\sigma }$ at t=0.6 and θ show the following relationships: ${\bar{D}}_{2}^{A}\left(t=0.6\right)=0.002-0.002\times \exp\left(-0.05\theta \right)$ and ${\bar{D}}_{2}^{B}\left(t=0.6\right)=0.003-0.002\times \exp(-0.05\theta$), respectively.

**Figure 7:**
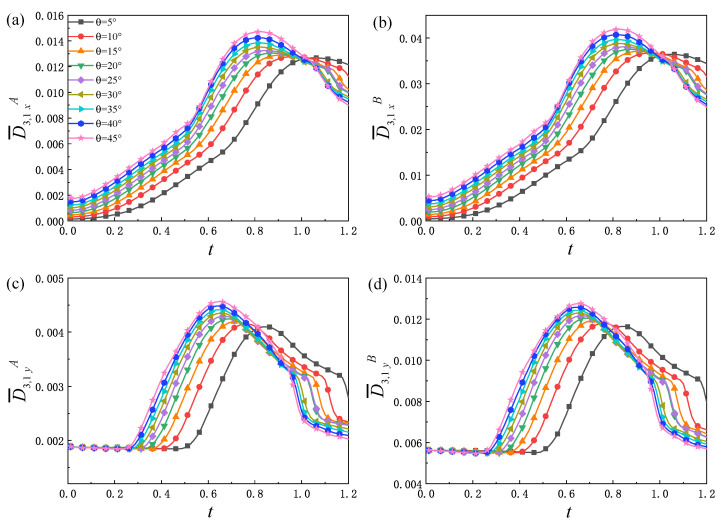
Evolution of the global average heat flux strength: (**a**) in the *x* direction ${\bar{D}}_{3,1x}^{A}$, (**b**) in the *x* direction ${\bar{D}}_{3,1x}^{B}$, (**c**) in the *y* direction ${\bar{D}}_{3,1y}^{A}$, and (**d**) in the *y* direction ${\bar{D}}_{3,1y}^{B}$, under different initial interface inclination angles.

The non-equilibrium quantities ${\bar{D}}_{3,1x}^{\sigma }$, ${\bar{D}}_{3,1y}^{\sigma }$ and ${\bar{D}}_{3,1}^{\sigma }$ related to heat conduction are investigated next. Figure 7 shows the variation trend of the non-equilibrium quantities ${\bar{D}}_{3,1x}^{\sigma }$, ${\bar{D}}_{3,1y}^{\sigma }$ under different θ with time. From Figure 7a,b, it can be observed that, for each θ, ${\bar{D}}_{3,1x}^{\sigma }$ increases and then decreases. In this process, there are three main physical mechanisms: (1) The interface is elongated and the contact area between the two components increases, which increases the heat flow between the two components. (2) The two components penetrate each other, which makes their particle density gradient near the material interface decrease, and the heat conduction rate with material diffusion decreases during this process. (3) The temperature field becomes uneven and constantly changing. The more obvious the temperature field changes, the greater the impact of the heat conduction. The above three physical mechanisms interact and compete with each other, making ${\bar{D}}_{3,1x}^{\sigma }$ show the trend of rising first and then falling.

**Figure 8:**
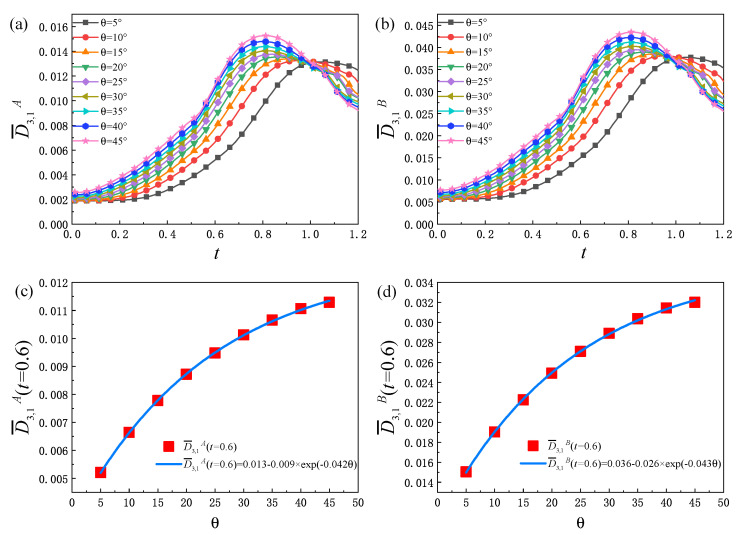
The evolution of the non-equilibrium quantity ${\bar{D}}_{3,1}^{\sigma }$ of components *A* (**a**) and *B* (**b**) under different initial interface inclination angles, the relationship between the value of the ${\bar{D}}_{3,1}^{A}$ (**c**) and ${\bar{D}}_{3,1}^{B}$ (**d**) at t=0.6 and θ.

Moreover, as can be seen from Figure 7c,d, for different θ, ${\bar{D}}_{3,1y}^{\sigma }$ remains almost constant in the early stage. This is because the width of the transition layer increases slowly, and there is no obvious vortex structure in the fluid system at this stage, and the spatial distribution of temperature has little variation in the *y* direction; therefore, ${\bar{D}}_{3,1y}^{\sigma }$ is maintained near a fixed value. Then, with the evolution of time, the nonlinearity of the fluid system and the contact area of the two components increase, which leads to an increase in the heat exchange, so ${\bar{D}}_{3,1y}^{\sigma }$ increases greatly. In the later stage, the mixing degree of the two components is further deepened, and the physical quantity gradient decreases, which makes ${\bar{D}}_{3,1y}^{\sigma }$ decrease. Under the combined action of ${\bar{D}}_{3,1x}^{\sigma }$ and ${\bar{D}}_{3,1y}^{\sigma }$, ${\bar{D}}_{3,1}^{\sigma }$ has a similar change trend as shown in Figure 8a,b. Consequently, the evolution law of ${\bar{D}}_{3,1}^{\sigma }$ can be obtained through the analysis of ${\bar{D}}_{3,1x}^{\sigma }$ and ${\bar{D}}_{3,1y}^{\sigma }$. Figure 8c,d shows the relationship of ${\bar{D}}_{3,1}^{\sigma }\left(t=0.6\right)$ and θ. The specific expressions are ${\bar{D}}_{3,1}^{A}\left(t=0.6\right)=0.013-0.009\times \exp\left(-0.042\theta \right)$ and ${\bar{D}}_{3,1}^{B}\left(t=0.6\right)=0.036-0.026\times \exp(-0.043\theta$), respectively. In addition, from Figure 7 and Figure 8, it can be seen that, before reaching the peak, ${\bar{D}}_{3,1x}^{\sigma }$, ${\bar{D}}_{3,1y}^{\sigma }$ and ${\bar{D}}_{3,1}^{\sigma }$ increase with the increase in the initial interface inclination angle.

**Figure 9:**
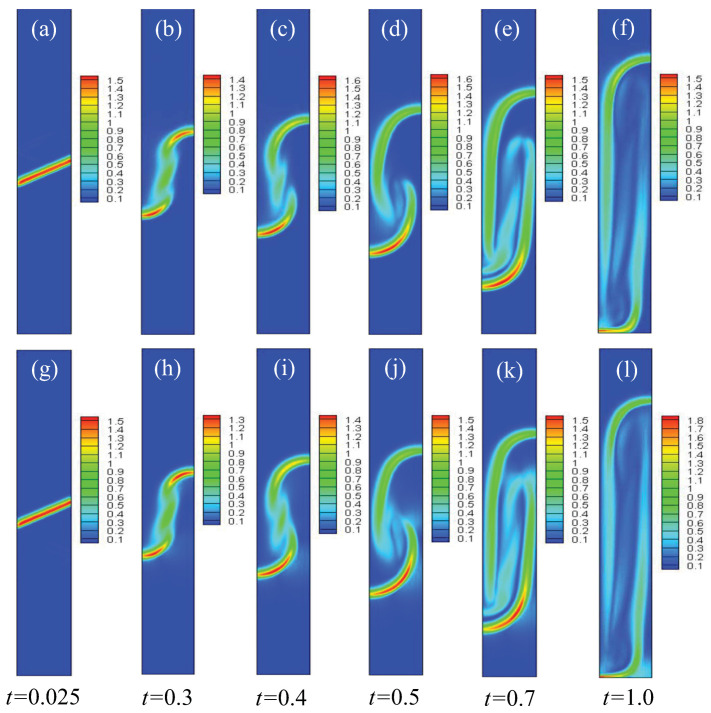
Contours of the global TNE strength in the case of θ=30° at times *t* = 0.025, 0.3, 0.4, 0.5, 0.7, and 1.0, respectively. Parts (**a**–**f**) are for component σ=*A* and (**g**–**l**) are for σ = *B*.

To further deepen our understanding of the TNE effects of the RT instability from a global perspective, the global average TNE strength ${\bar{D}}^{\sigma }$ is discussed. In order to obtain an intuitive understanding, the contours of the spatial distribution of the non-equilibrium region in the evolution process of the RT instability are shown in Figure 9. It can be observed that the non-equilibrium strength near the material interface is largest; this is because the physical gradients towards the interface are sharpest. With the development of the RT instability, the material interface is elongated, the contact area of the two components increases, and many small structures appear in the system, which results in the increasing area of the non-equilibrium region. Later, as a result of dissipation and/or mutual penetration of two components, the small structures gradually disappear and the physical gradients become smooth.

**Figure 10:**
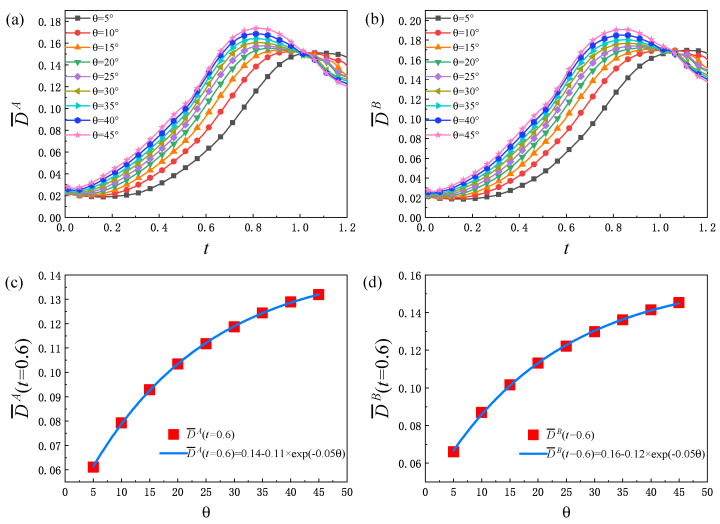
The global average TNE quantity ${\bar{D}}^{\sigma }$ of components *A* (**a**) and *B* (**b**) versus time under different initial interface inclination angles, the fitting curve of the value of ${\bar{D}}^{A}$ (**c**) and ${\bar{D}}^{B}$ (**d**) at t=0.6 and the initial interface inclination angle.

Figure 10 displays the evolution of the global average TNE intensity ${\bar{D}}^{\sigma }$. From Figure 10a,b, it can be found that, for each θ, ${\bar{D}}^{\sigma }$ increases first and then decreases, and increases with the increase in θ. The combined effect of two physical mechanisms leads to the trend of ${\bar{D}}^{\sigma }$ increasing first and then decreasing. One effect is that the fluid structure becomes more and more complex with time, and the contact area of the two components increases, so the non-equilibrium region continues to increase. Another effect is that, due to the diffusion and dissipation, the density, velocity and other macroscopic physical quantities of the two components decrease. In general, during the process of evolution, the increase in the non-equilibrium region enhances the global average non-equilibrium strength, while the decrease in the macroscopic physical gradient leads to a decrease in the global average non-equilibrium strength. In the early stage, the increase in the non-equilibrium region plays a leading role in the development of ${\bar{D}}^{\sigma }$, which leads to an increasing in ${\bar{D}}^{\sigma }$. In the later period, with the decrease in the macroscopic physical quantity gradient of the two components, ${\bar{D}}^{\sigma }$ decreases. In addition, as displayed in Figure 10c,d, the exponential relationships between the global average TNE quantity ${\bar{D}}^{\sigma }$ at t=0.6 and the initial interface inclination angle θ are as follows: ${\bar{D}}^{A}\left(t=0.6\right)=0.14-0.11\times \exp\left(-0.05\theta \right)$ and ${\bar{D}}^{B}\left(t=0.6\right)=0.16-0.12\times \exp\left(-0.05\theta \right)$, respectively.

**Figure 11:**
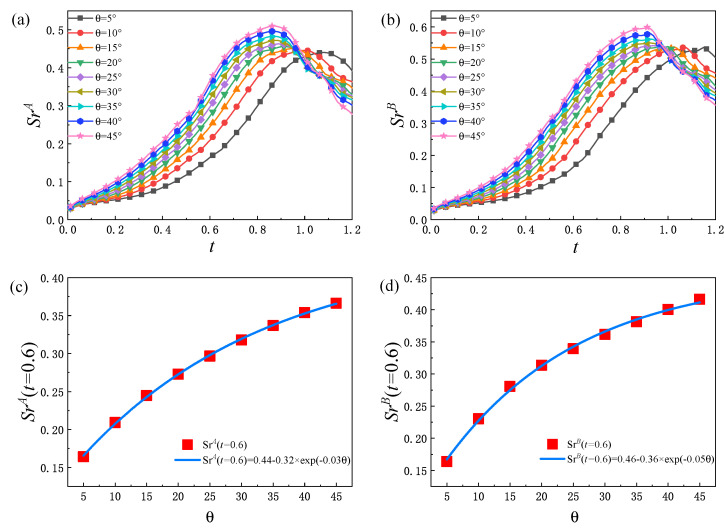
Evolution of the proportion of the non-equilibrium region ${Sr}^{A}$ of component *A* (**a**) and ${Sr}^{B}$ of component *B* (**b**), relationship between the value of ${Sr}^{A}$ (**c**) and ${Sr}^{B}$ (**d**) at t=0.6 and the initial interface inclination angle.

In order to further analyze the global average non-equilibrium strength of the system, the non-equilibrium region ratio ${Sr}^{\sigma }$ is studied. Here, ${Sr}^{\sigma }$ is equal to the ratio of the non-equilibrium area occupied by the component σ to the total area of the system. Figure 11a,b delineates the evolution of the non-equilibrium region ratio ${Sr}^{\sigma }$, from which it can be found that ${Sr}^{\sigma }$ increases first and then decreases with time. Moreover, ${Sr}^{\sigma }$ increases with the increase in θ before ${Sr}^{\sigma }$ reaches the peak. In the early stage, with the evolution of the fluid system, the interface between the two components is continuously elongated, and the non-equilibrium region of each component increases, so that ${Sr}^{\sigma }$ rises. In the later stage, as the two components are fully mixed, the small structures in the fluid system gradually disappear under the impact of diffusion and dissipation, and the gradients of various physical quantities are smoothed, which makes ${Sr}^{\sigma }$ show a downward trend. From Figure 11c,d, it can be further found that there is a functional relationship between the value of the non-equilibrium region ratio ${Sr}^{\sigma }$ at t=0.6 and the initial interface inclination angle θ, as follows: ${Sr}^{A}\left(t=0.6\right)=0.44-0.32\times \exp\left(-0.03\theta \right)$ and ${Sr}^{B}\left(t=0.6\right)=0.46-0.36\times \exp\left(-0.05\theta \right)$ increases exponentially with the increase in θ. Physically, the larger θ is, the faster the system develops, and the larger the non-equilibrium region in the early stage.

## 4. Conclusions

In this paper, the effect of the inclination angle on the inclined interface compressible RT instability is studied using the two-component DBM. Firstly, the evolution trend of global average density gradients $|\bar{\nabla_{x}\rho }|$, $|\bar{\nabla_{y}\rho }|$ and $|\bar{\nabla \rho }|$ are analyzed. In general, $|\bar{\nabla_{x}\rho }|$ displays a trend of increasing first and then decreasing. Both $|\bar{\nabla_{y}\rho }|$ and $|\bar{\nabla \rho }|$ decrease first, then increase, and finally decrease. In addition, the larger the initial interface inclination angle is, the faster the fluid system evolves, and the larger the density gradient is.

Next, the TNE behaviors are explored during the RT process. Three kinds of non-equilibrium quantities are investigated. (1) The non-equilibrium quantity ${\bar{D}}_{2}^{\sigma }$ decreases first, then increases and finally decreases, and ${\bar{D}}_{2}^{\sigma }$ increases with the increase in θ. (2) In general, the non-equilibrium quantities ${\bar{D}}_{3,1x}^{\sigma }$, ${\bar{D}}_{3,1y}^{\sigma }$ and ${\bar{D}}_{3,1}^{\sigma }$ related to heat conduction increase first and then decrease with time, and increase with the increase in θ. Physically, there are three competition mechanisms. In the first, during the RT process, the interface is elongated and the contact area between the two components increases, which increases the heat flux between the two components. The second is that with the evolution of time, the two components penetrate each other, which makes their density gradient near the material interface decrease, and the heat conduction rate with material diffusion decreases during this process. In the third, the temperature field becomes uneven and constantly changing. The more significant the temperature field changes, the greater the heat conduction changes. (3) The global average non-equilibrium strength ${\bar{D}}^{\sigma }$ increases first and then decreases, and it increases with the growth of θ. Physically, there are two competitive mechanisms in the RT process. One is that with the evolution of the fluid system, the fluid structure becomes more and more complex, and the contact area of the two components increases, which causes the non-equilibrium region to increase. Another is that due to the diffusion and dissipation, the density, velocity and other macroscopic physical quantities of the two components decrease. In general, during the RT process, the increase in the non-equilibrium region enhances the global average non-equilibrium strength ${\bar{D}}^{\sigma }$, while the decrease in the macroscopic physical gradient leads to a decrease in ${\bar{D}}^{\sigma }$.

Lastly, the proportion of non-equilibrium region ${Sr}^{\sigma }$ is discussed. It is found that ${Sr}^{\sigma }$ increases first and then decreases with time. The reason for this phenomenon is that the interface of the two components is continuously elongated with the evolution of the fluid system, which leads to the increase in ${Sr}^{\sigma }$. Due to the diffusion and dissipation, the small structure of the fluids in the system gradually disappears, and the physical quantity gradient decreases, so the ${Sr}^{\sigma }$ decreases in the latter. These results can help us to understand the physical mechanism of the compressible RT instability from a kinetic perspective.

## Figures and Tables

**Figure A1:**
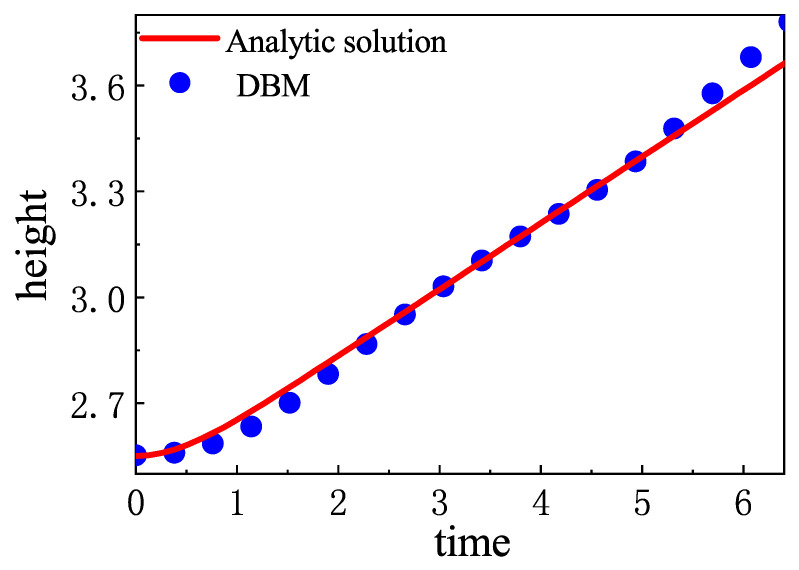
The height of the bubble front versus time. The circular symbols represent the DBM result and the solid red line denotes the analytical solution.

**Figure A2:**
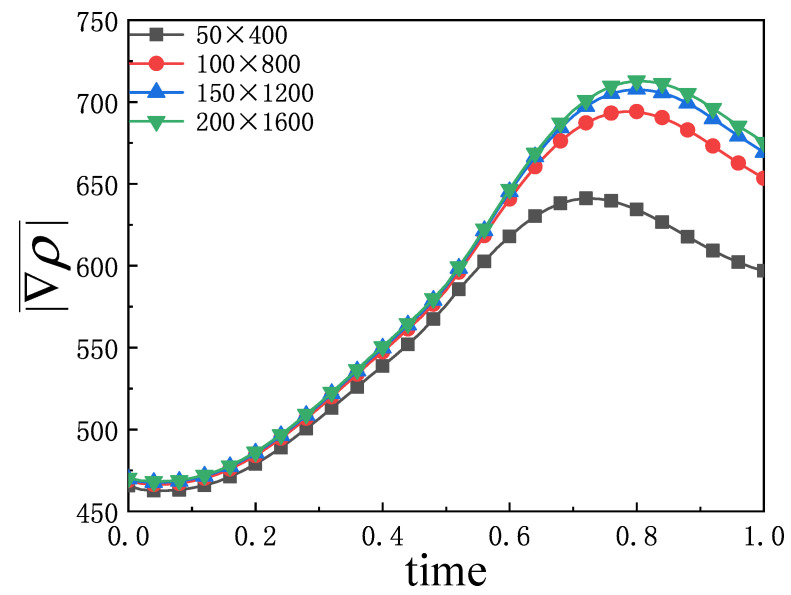
Grid convergence test of simulations of the RT instability: the global average density gradient evolves over time with various mesh grids.

## Data Availability

Data are contained within the article.

## Appendix A

To verify the accuracy of the two-component DBM, we compare the DBM simulation result with the analytical solution given in Ref. [47]. The initial configuration is given in Ref. [42]. Figure A1 plots the height of the bubble front versus time. The circular symbols represent the DBM calculation result with Re=500, and the solid red line denotes the analytical solution. Clearly, the result of the DBM is roughly in agreement with the analytical solution. It should be noted that there is a small difference between the current simulation result and the analytical solution given in Ref. [47] because the DBM considers viscosity, compressibility, and rotation, while the analytical solution in Ref. [47] is obtained under the assumption of inviscosity, incompressibility, and irrotation. Therefore, the DBM simulation results are more accurate than the analytical solutions.

## Appendix B

The grid convergence test is an important index by which to judge the reliability of numerical simulation results. In order to verify the validity of the simulation results, different grids are used to analyze the grid convergence. The RT instability with θ=30° is simulated in the region of $L_{x}\times L_{y}=0.025\times 0.2$. In Figure A2, the four different symbolic lines show the simulation results of the density gradient under four different grids $N_{x}\times N_{y}=50\times 400$, 100×800, 150×1200, 200×1600, respectively. It can be seen that, the simulation results are converging with the refinement of the grid. Therefore, taking into account the numerical accuracy, the mesh 200×1600 is selected in this paper, and the corresponding space step is $\Delta x\;\left(=\;\Delta y\;\right)=\;1.25\times 10^{-4}$.
